# Supplementary figures and images for: Neuronal death in pneumococcal meningitis is triggered by pneumolysin and RrgA interactions with β-actin
Source: PLoS Pathog. 2021 Mar 24;17(3):e1009432. doi: 10.1371/journal.ppat.1009432 (PMC7990213; doi:10.1371/journal.ppat.1009432)

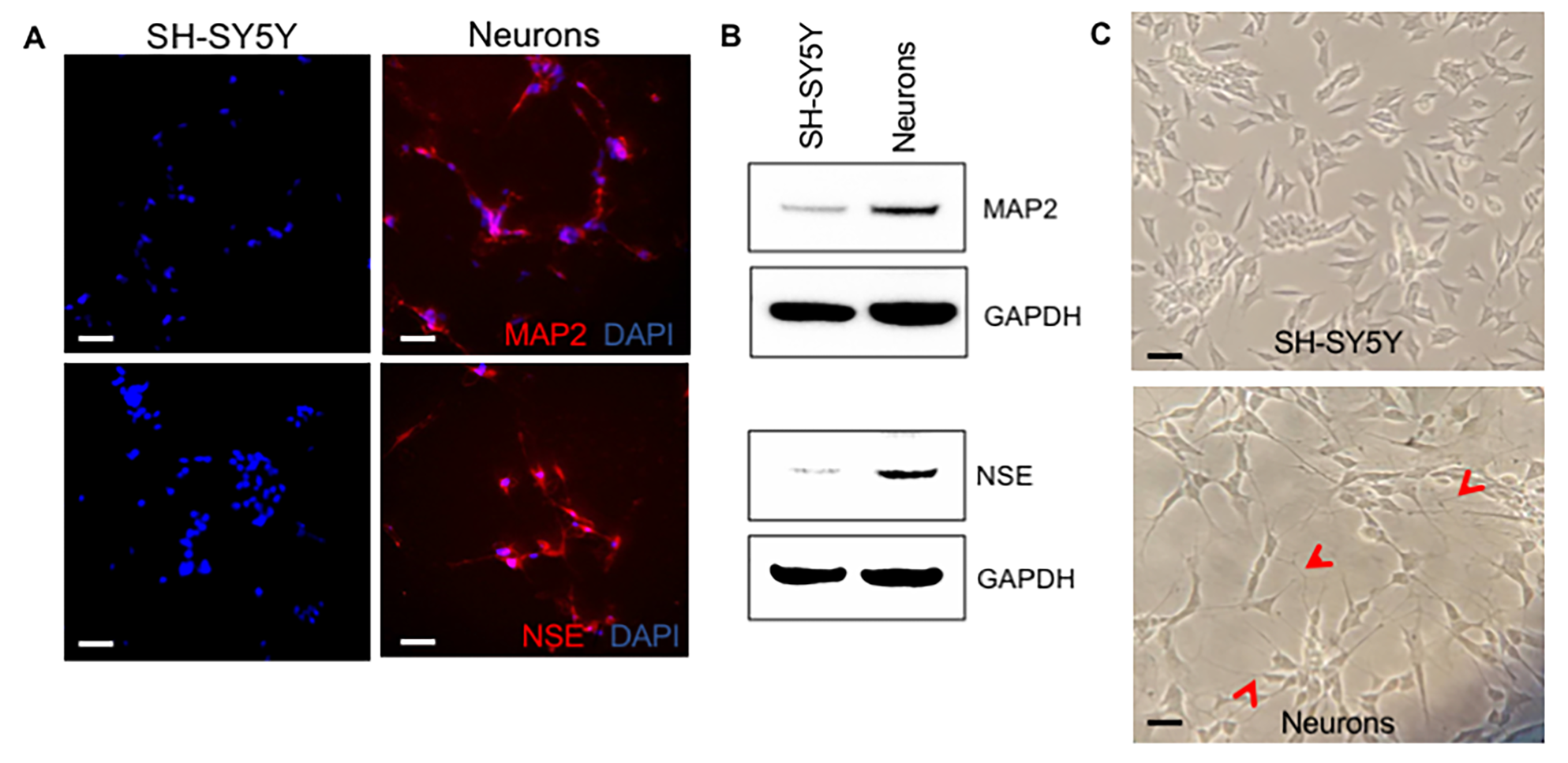

Supplement: S1 Fig — Generation of mature differentiated neurons from human SH-SY5Y neuroblastoma cells was assessed by the expression of neuronal specific markers MAP2 and NSE via (A) immunofluorescence microscopy and (B) western blot analysis in which GAPDH was used as loading control. White scale bars in S1A Fig represent 100 μm, in the blot in S1B Fig the same protein concentration of both SH-SY5Y and neurons was loaded into the SDS-page gel. (C) Through phase contrast light microscopy analysis, we also observed that differentiated neurons displayed the neuronal typical cell-to-cell connections and axon formation (red arrows), black scale bars represent 100 μm. (TIF) [file ppat.1009432.s001.tif]

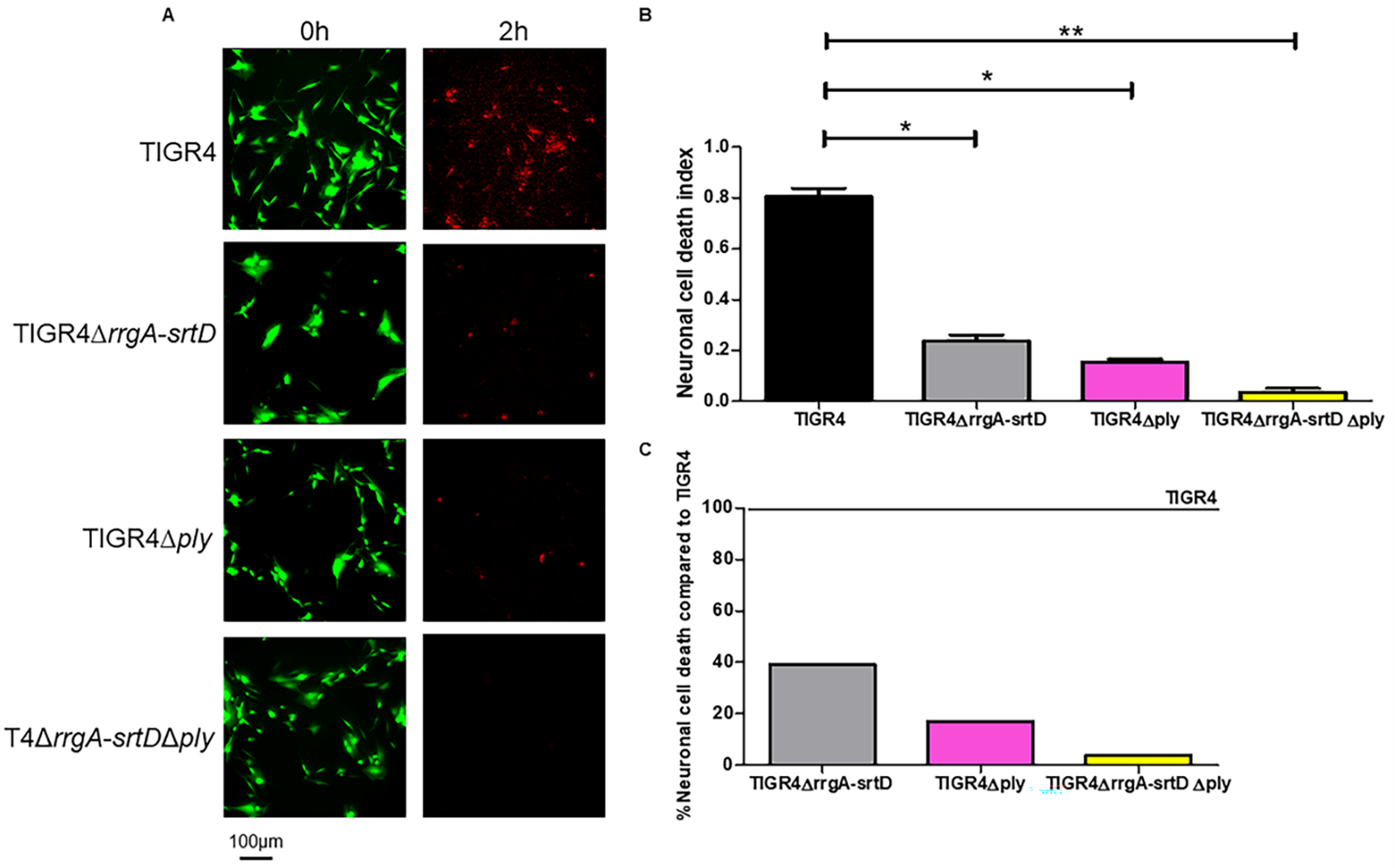

Supplement: S2 Fig — (A) Images from live-cell imaging at the start (time 0) and at the end, 2 hours post-infection. Differentiated neurons stained with LIVE/DEAD dye express green fluorescence when they are alive, and a red fluorescence when undergoing cell death. (B) Quantification of the neuronal cell death in the 2-hour infection experiment shown in Fig 1A; Green (488 nm) / Red (594 nm) represents the neuronal cell death index, calculated by dividing the total area occupied by the green fluorescence signal at time 0 by the total area occupied by the red fluorescence signal at the end of the infection. Per each pneumococcal strain, a total of 2 biological replicates (2 wells with neurons, each well seeded in a different day) have been used for the 1-hour experiment, and a total of 2 biological replicates (2 wells with neuron, each well seeded in a different day). Columns in the graphs represent average values, error bars represent standard deviations. ** = p<0.001, * = p<0.05. (C) Percentage average values of neuronal cell death calculated setting the average value of neuronal cell death of TIGR4 to 100%. The percentage average values were calculated using the neuronal cell death index values shown in Fig 1B. (TIF) [file ppat.1009432.s002.tif]

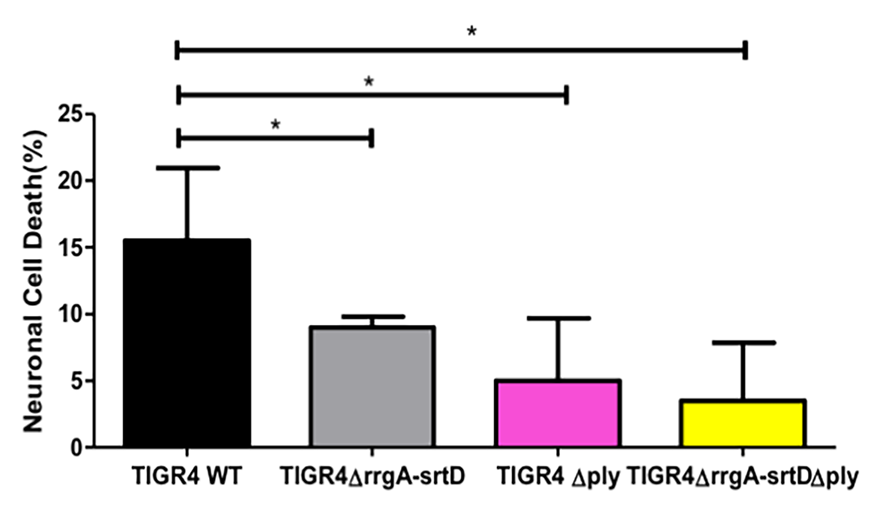

Supplement: S3 Fig — Neuronal cell death measured by analysis of LDH release in neurons infected with TIGR4, TIGR4ΔrrgA-srtD, TIGR4Δply, and TIGR4ΔrrgA-srtDΔply. Columns represent average values of LDH release, error bars represent standard deviation values calculated among three biological replicates (each biological replicated included two technical replicates); * = p<0.05. (TIF) [file ppat.1009432.s003.tif]

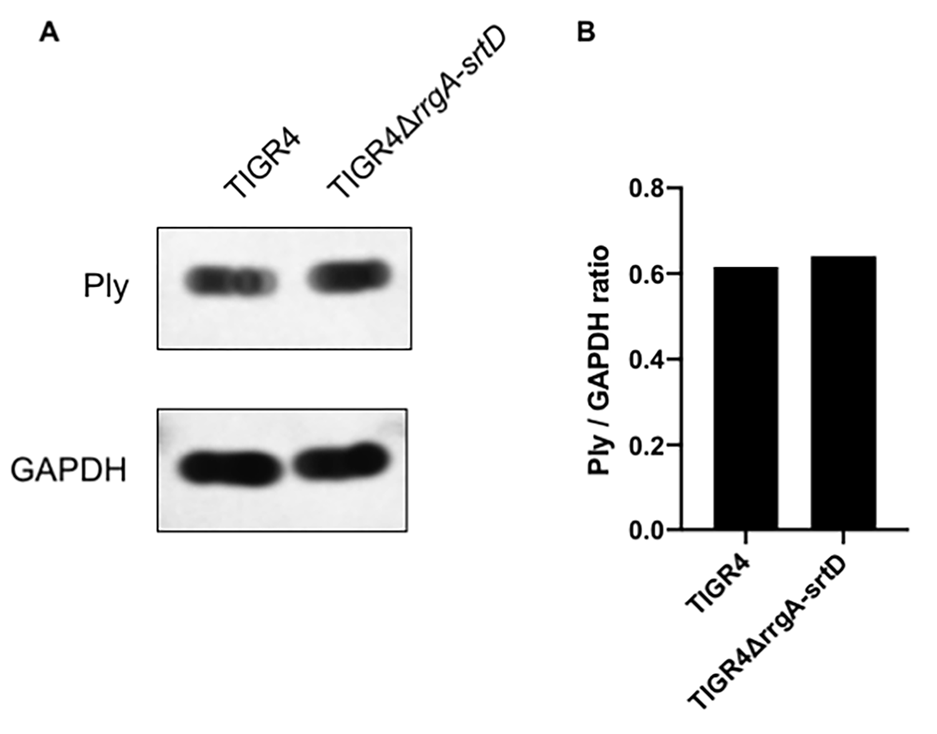

Supplement: S4 Fig — (A) Western blot analysis showing similar expression of Ply in TIGR4 and TIGR4ΔrrgA-srtD; for both bacterial strains, the same protein content was loaded in the SDS-page. (B) Quantification of Ply expression in TIGR4 and TIGR4ΔrrgA-srtD calculated by dividing the intensity of Ply bands per the intensity of the GAPDH (loading control) bands; band intensity values were measured with Image J. (TIF) [file ppat.1009432.s004.tif]

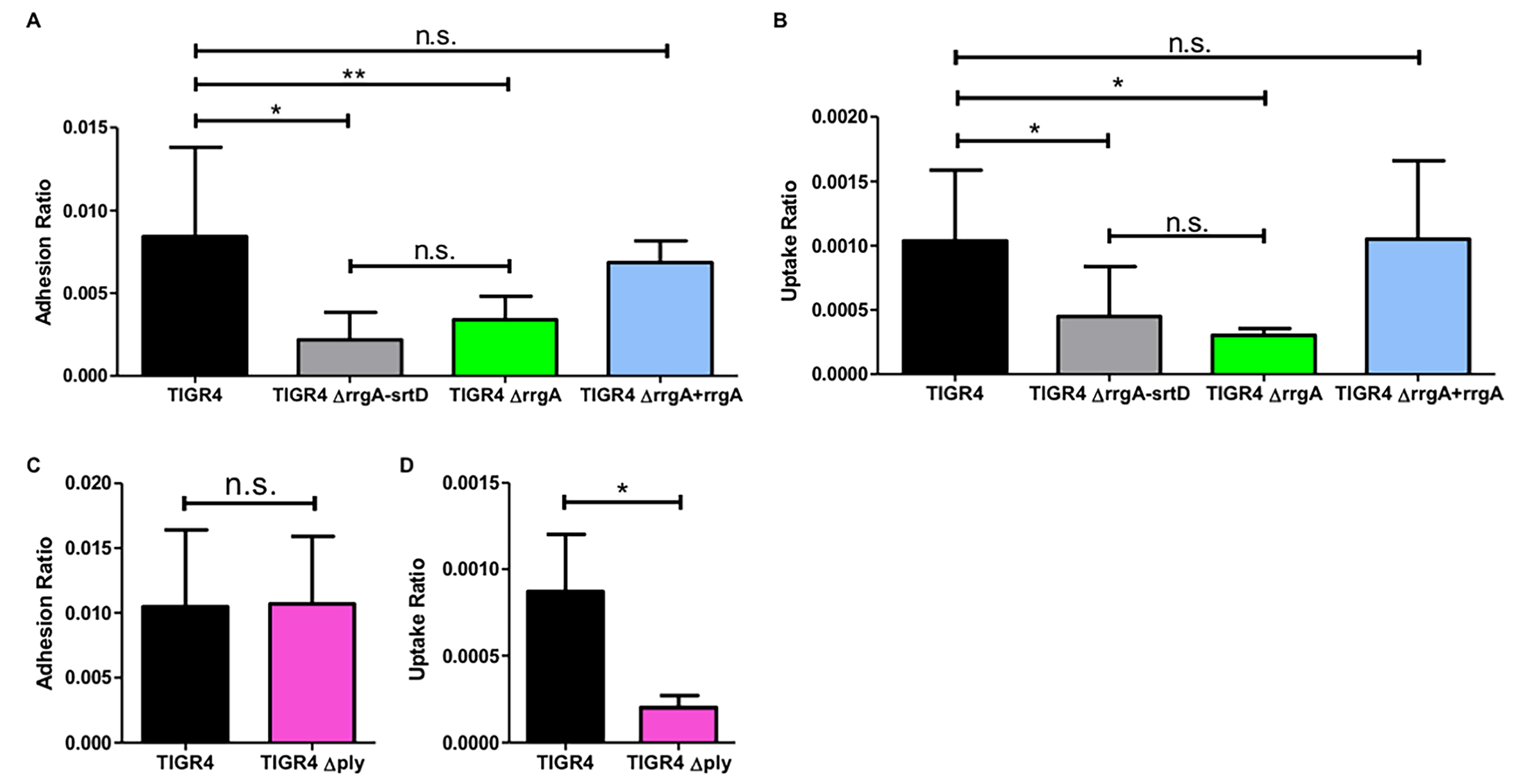

Supplement: S5 Fig — SH-SY5Y cells were challenged with pneumococci of MOI 10 and after 2 hours (A) adhesion to and (B) invasion of neuronal cells were measured. Strains used were wt TIGR4 and its isogenic mutants in the pilus, TIGR4ΔrrgA-srtD, rrgA, TIGR4ΔrrgA, and the rrgA mutant complemented with rrgA. TIGR4ΔrrgA+rrgA. (C) Adhesion and (D) invasion were measured using wt TIGR4 and its isogenic mutant in ply, TIGR4Δply. Adhesion ratio was calculated by dividing the total number of bacteria in each well for each pneumococcal strain after 2 hours infection by the total number of adhered bacteria in each well for each pneumococcal strain. For all graphs (A-D) the columns represent average values, and error bars represent standard deviations. Each graph shows data from at least three (n≥3) biological replicates. *** = p<0.0001, ** = p<0.001, ** = p<0.05, n.s. = not-significant. (TIF) [file ppat.1009432.s005.tif]

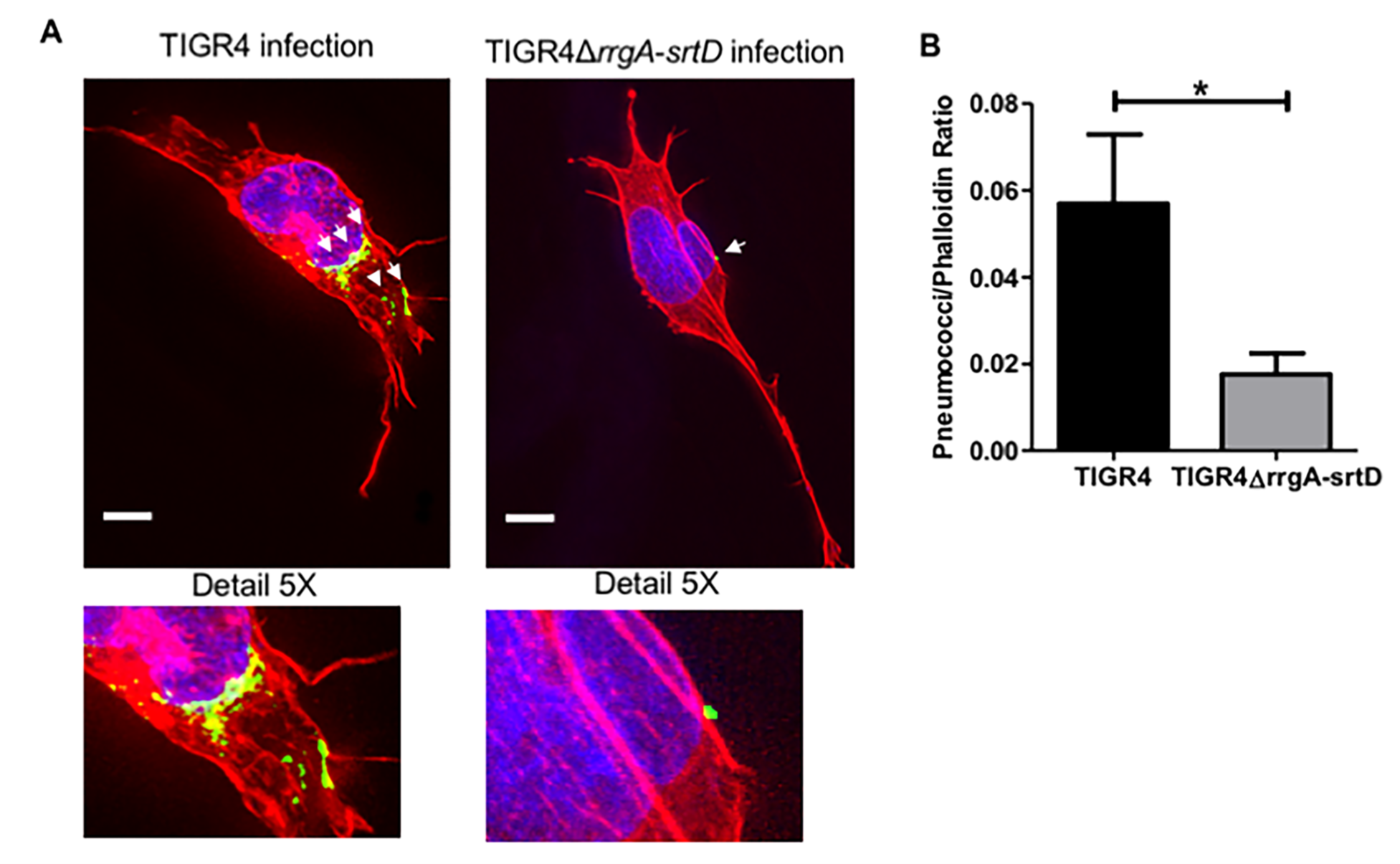

Supplement: S6 Fig — (A) High-resolution fluorescence microscopy was used to visualize piliated and non-piliated pneumococci that adhered to SH-SY5Y cells. Neurons were stained with Phalloidin (red), and TIGR4 and TIGR4ΔrrgA-srtD were stained with anti-serotype 4 capsule antibody combined with goat anti rabbit Alexa Fluor 488 (green). White arrows point to pneumococci that adhered to SH-SY5Y cells. White scale bars represent 10 μm. The images shown are two representative images selected among 200 cells with adhered bacteria imaged per pneumococcal strain. The panel “Detail 5X” displays a 5X-magnified image of the area in the original images with bacteria that adhered to neurons. (B) Quantification of the number of bacteria that adhered to neurons based on the microscopy analysis results shown in S1A Fig. For quantification, the bacterial fluorescence signal on SH-SY5Y cells, in each image (n = 200 SH-SY5Y cells with adhered bacteria, per each pneumococcal strain) the area occupied by the green fluorescence signal of the bacteria, was divided by the area occupied by the red fluorescence signal of SH-SY5Y cells. All areas were measured in square pixels and calculated with the software Image J. The Pneumococci/Phalloidin ratio is shown on the Y axis. Columns in the graph represent average values, error bars represent standard deviations, * = p<0.05. (TIF) [file ppat.1009432.s006.tif]

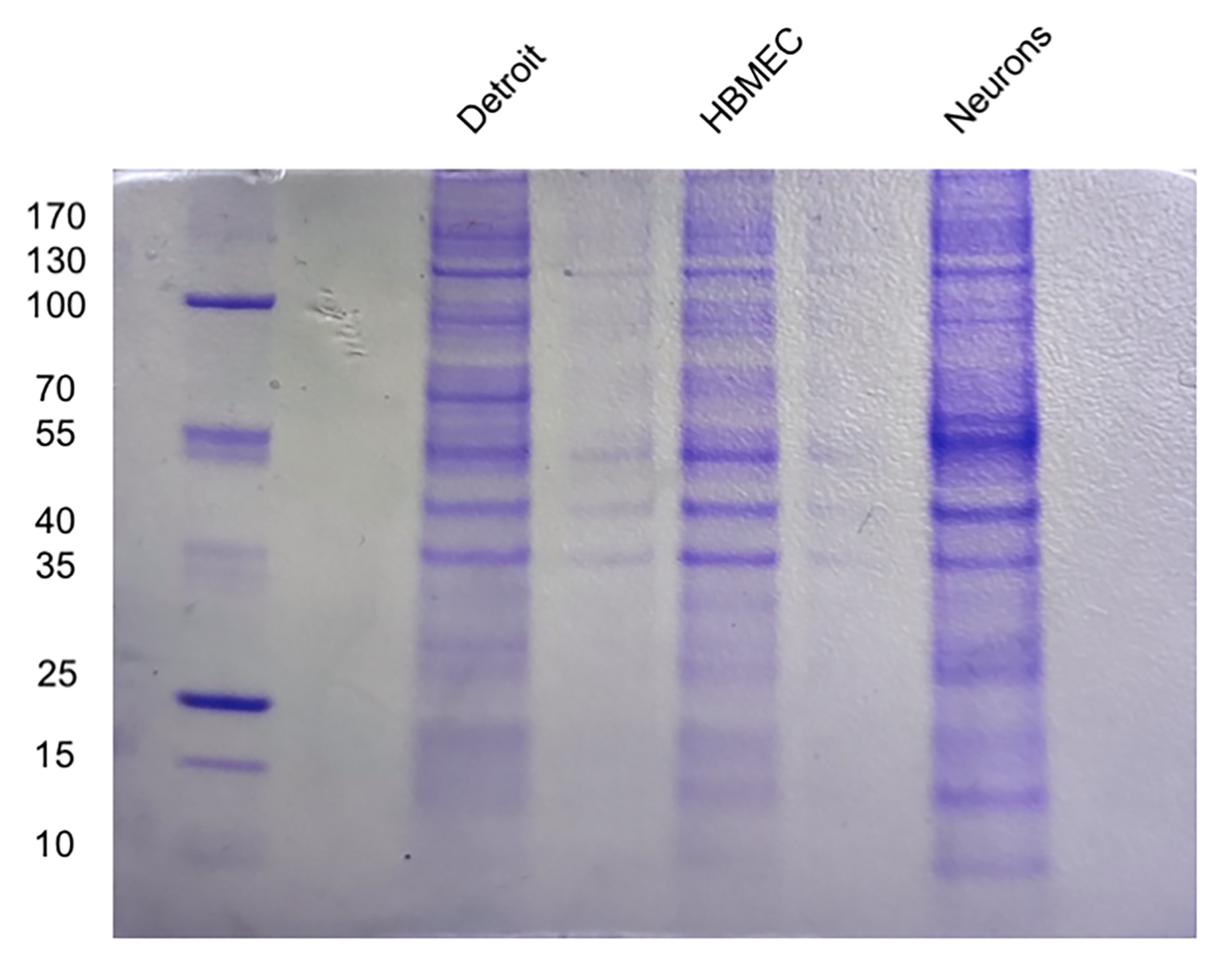

Supplement: S7 Fig — Before performing the co-immunoprecipitation experiments, the quality of the cell lysate of differentiated neurons was assessed by SDS-page electrophoresis and Coomassie staining. The clear detection of the neuronal protein bands ranging from low to high molecular sizes suggested good quality of the cell lysate of HBMEC, Detroit and neurons. The numbers on the left side of the image show the protein molecular weight in kDa. (TIF) [file ppat.1009432.s007.tif]

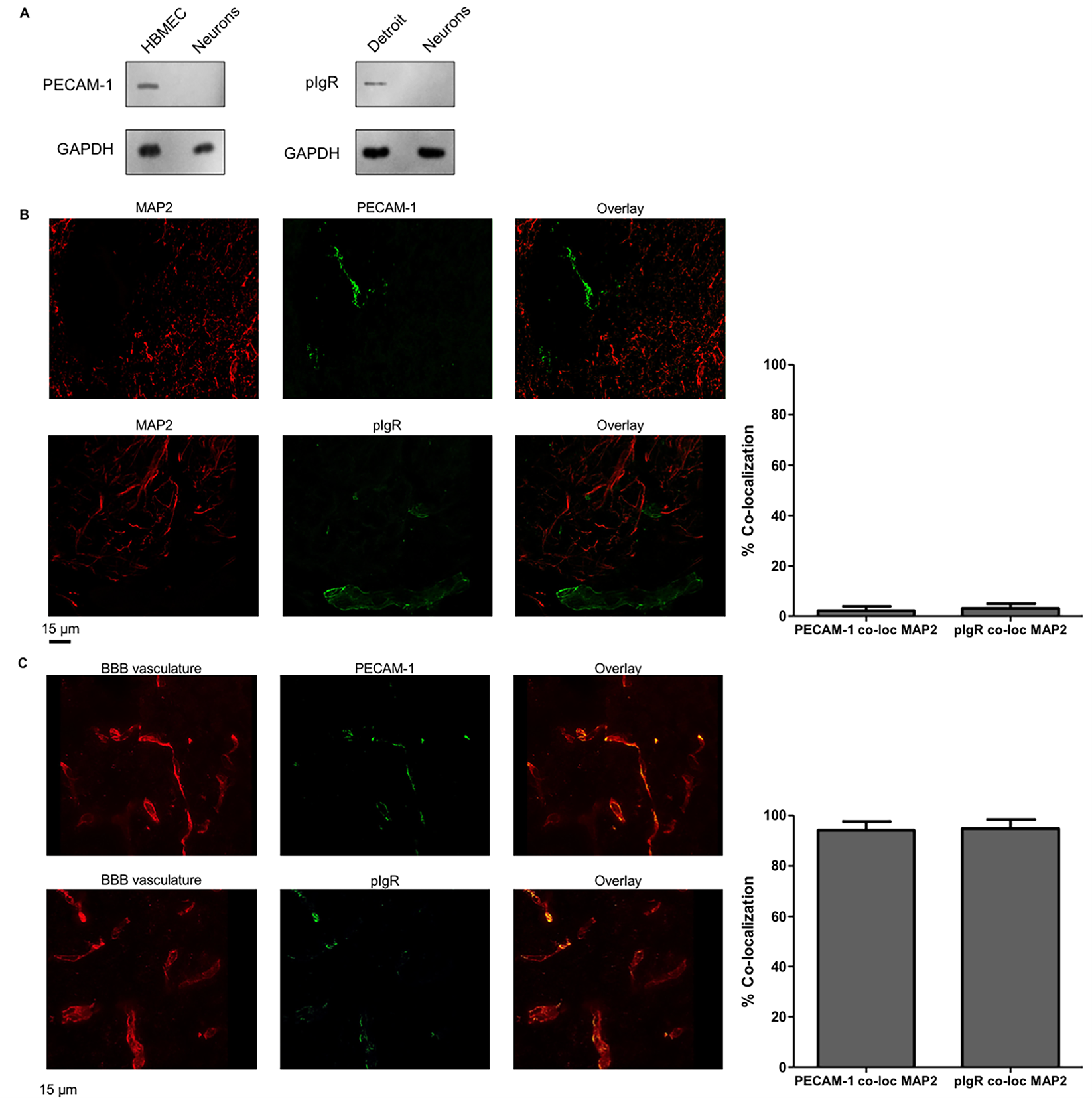

Supplement: S8 Fig — (A) Detection of PECAM-1 and pIgR in neurons by western blot analysis; HBMEC were used as positive control for PECAM-1 expression, Detroit were used as positive control for pIgR expression, GAPDH was used as loading control. (B) Immunofluorescence microscopy analysis using mouse brain tissue sections showing a co-staining of neurons (in red, stained for the neuronal marker MAP2), and PECAM-1 and pIgR (in green); the overlay and the quantification graph (Y axis shows the % co-localization between PECAM-1/pIgR signals with MAP2 neuronal signal) show that neither PECAM-1 nor pIgR fluorescent signals co-localize with neuronal signal. (C) Immunofluorescence microscopy analysis using mouse brain tissue sections showing a co-staining of brain vasculature (in red) and PECAM-1 and pIgR (in green); the overlay and the quantification graph (Y axis shows the % co-localization between PECAM-1/pIgR signals with vascular endothelium signal) show that PECAM-1 and pIgR fluorescent signals mainly co-localize with the vascular endothelium signal. For quantification graphs in B and C, nine mouse brain tissue sections were analyzed, and ten random images were taken per each section; the area occupied by the green fluorescence signal of PECAM-1 and pIgR, and the area occupied by the red fluorescent signal of vascular endothelium (lectin) and neurons (MAP2) were assessed for co-localization using the Co-loc function of Image J, and co-localization area were finally measured. was divided by the total area occupied neurons imaged through the DIC channel; columns in the graphs in B and C show average values among all images of all sections imaged per group. All areas were measured in square pixels and calculated with the software Image J. (TIF) [file ppat.1009432.s008.tif]

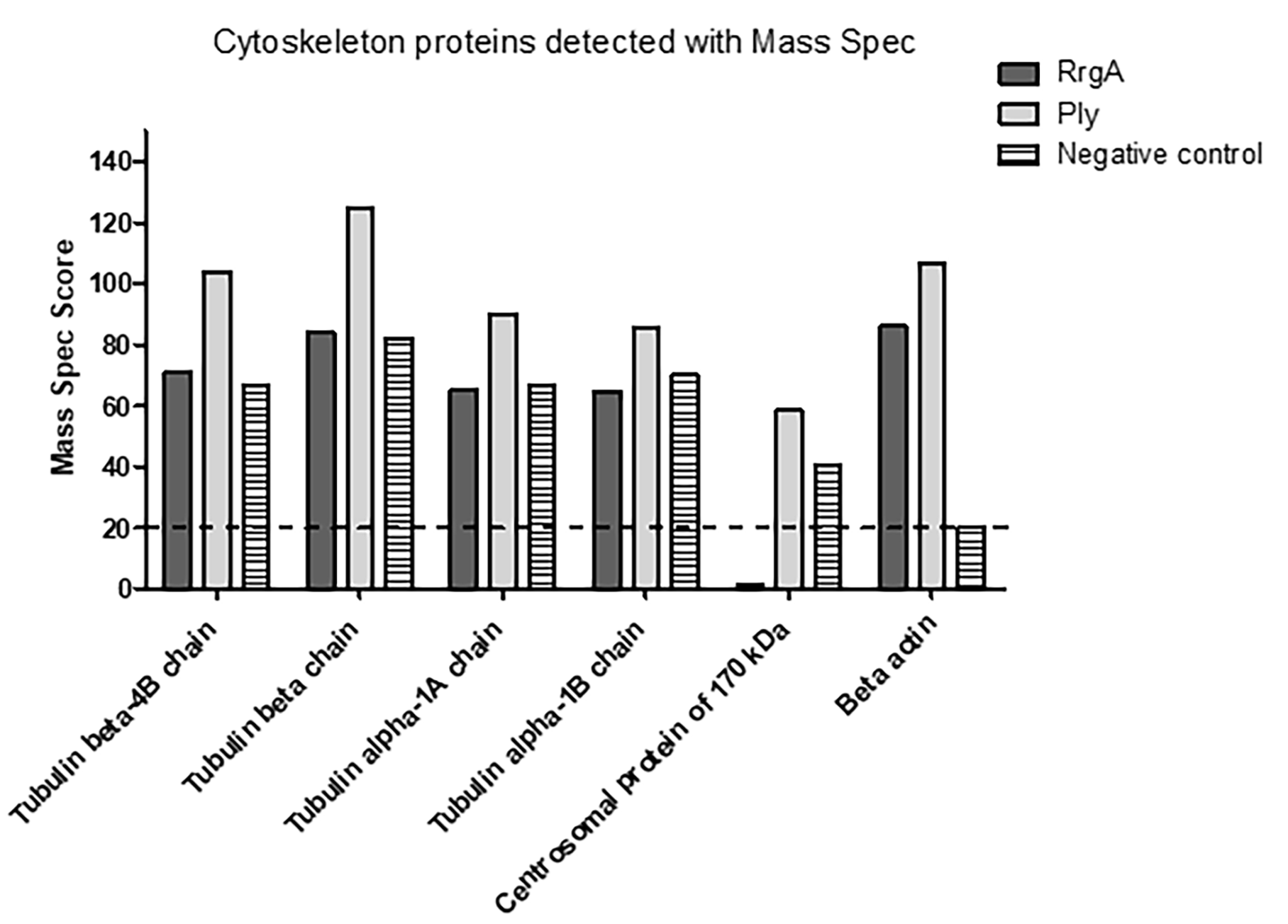

Supplement: S9 Fig — A pull-down assay was performed using differentiated neurons from SH-SY5Y cells and Ni-NTA beads-coupled-RrgA (RrgA), Ni-NTA beads-coupled-Ply (Ply) or Ni-NTA beads alone (Negative control) to identify proteins that bound to RrgA, or Ply respectively, as compared to beads alone. Mass spectrometry analysis identified the neuronal cytoskeleton proteins listed on the x axis and their presence is presented as Mass spectrometry scores on the Y axis. The dash black line at score = 20 represents the threshold of false positives. (TIF) [file ppat.1009432.s009.tif]

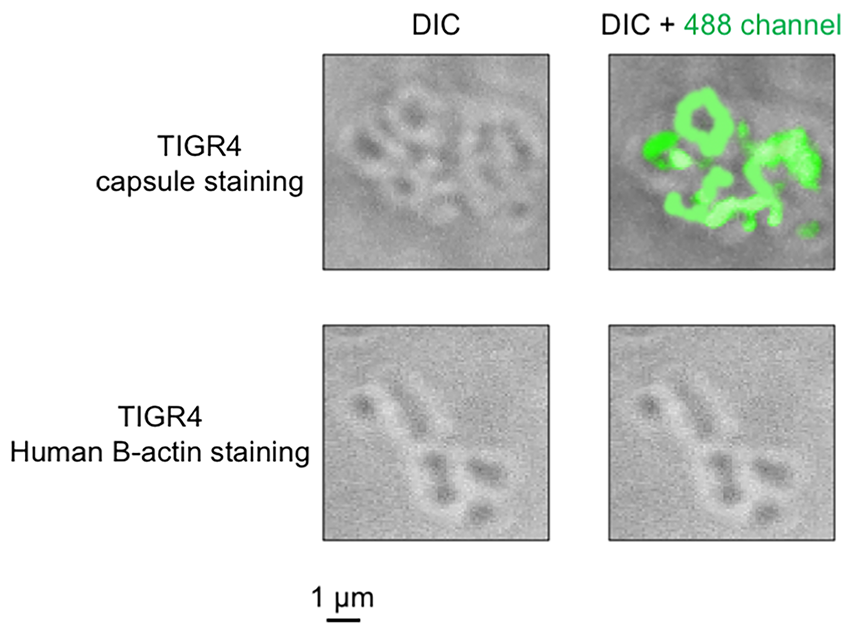

Supplement: S10 Fig — (A) Immunofluorescent microscopy analysis performed using TIGR4 pneumococci showing no β-actin fluorescent signal detected on the surface of the bacteria; (B) as positive control we used an anti-capsule serotype 4 antibody and the polysaccharide capsule was clearly detected around the bacterial cells. (TIF) [file ppat.1009432.s010.tif]

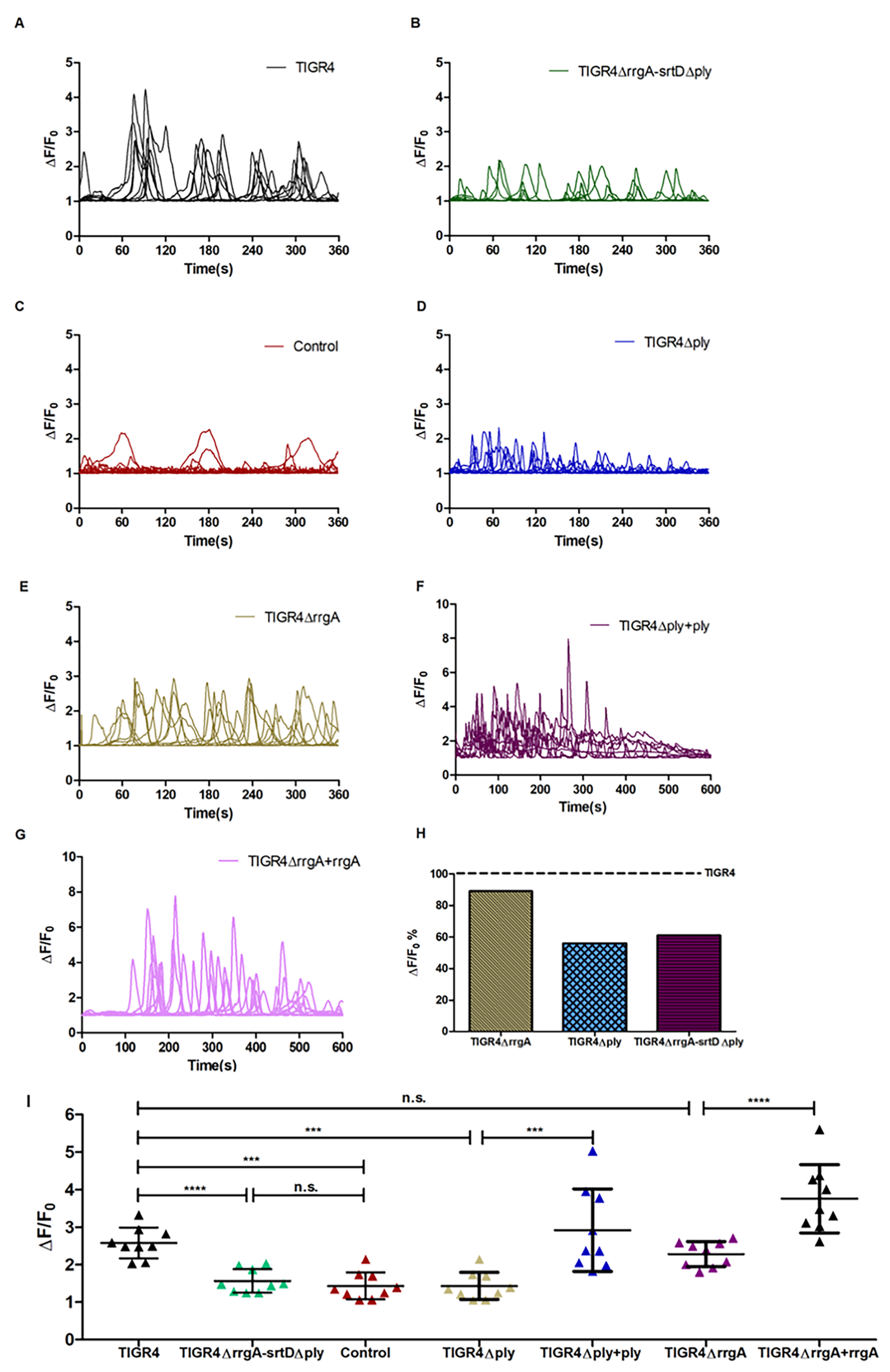

Supplement: S11 Fig — Intracellular Ca2+ release and influx in neurons infected with (A) TIGR4, (B)TIGR4ΔrrgA-srtDΔply, (C) non-infected neurons (Control), (D) TIGR4Δply and (E) TIGR4ΔrrgA were determined using Fluo-8 AM two hours post infection. Ca2+ imaging was performed for 6 minutes of time lapse in every 1 second using a FITC fluorescence channel. (F and G) Neurons were infected with TIGR4Δply (F) or TIGR4ΔrrgA (G), then 100ng/mL of Ply or RrgA was added 2 min prior to imaging and Ca2+ imaging was performed for 11 minutes of time lapse in every 1 second using FITC fluorescence channel. In A-G, the fluorescence intensity was measured using Image J from the region of interest (ROI) where a minimum of 10 ROI in each field was selected manually. ΔF/F0 was calculated from Image J-generated data (ΔF = Ft-F0, Ft represents the fluorescent intensity in each given time point and F0 represents the average fluorescent intensity of the resting value). Each graph displays the Ca2+ flux from 9 neuronal cells of 3 different experiments shown in 360 and 660 seconds. (H) Average ΔF/F0 values of relative peaks (n = 9) during 6 minutes within ROI were shown to compare intracellular Ca2+ levels; *** = p<0.0001, ** = p<0.001 n.s. = not-significant. Each data point in the graph represents one peak. (I) Percentage of average ΔF/F0 values of TIGR4ΔrrgA, TIGR4Δply and TIGR4ΔrrgA-srtDΔply compared to the ΔF/F0 values showed by neurons infected with wt TIGR4 (set to 100%). Each average value was calculated using the Ca2+ flux intensity values shown in Fig 7G (each dot in every group is one intensity value). (TIF) [file ppat.1009432.s011.tif]

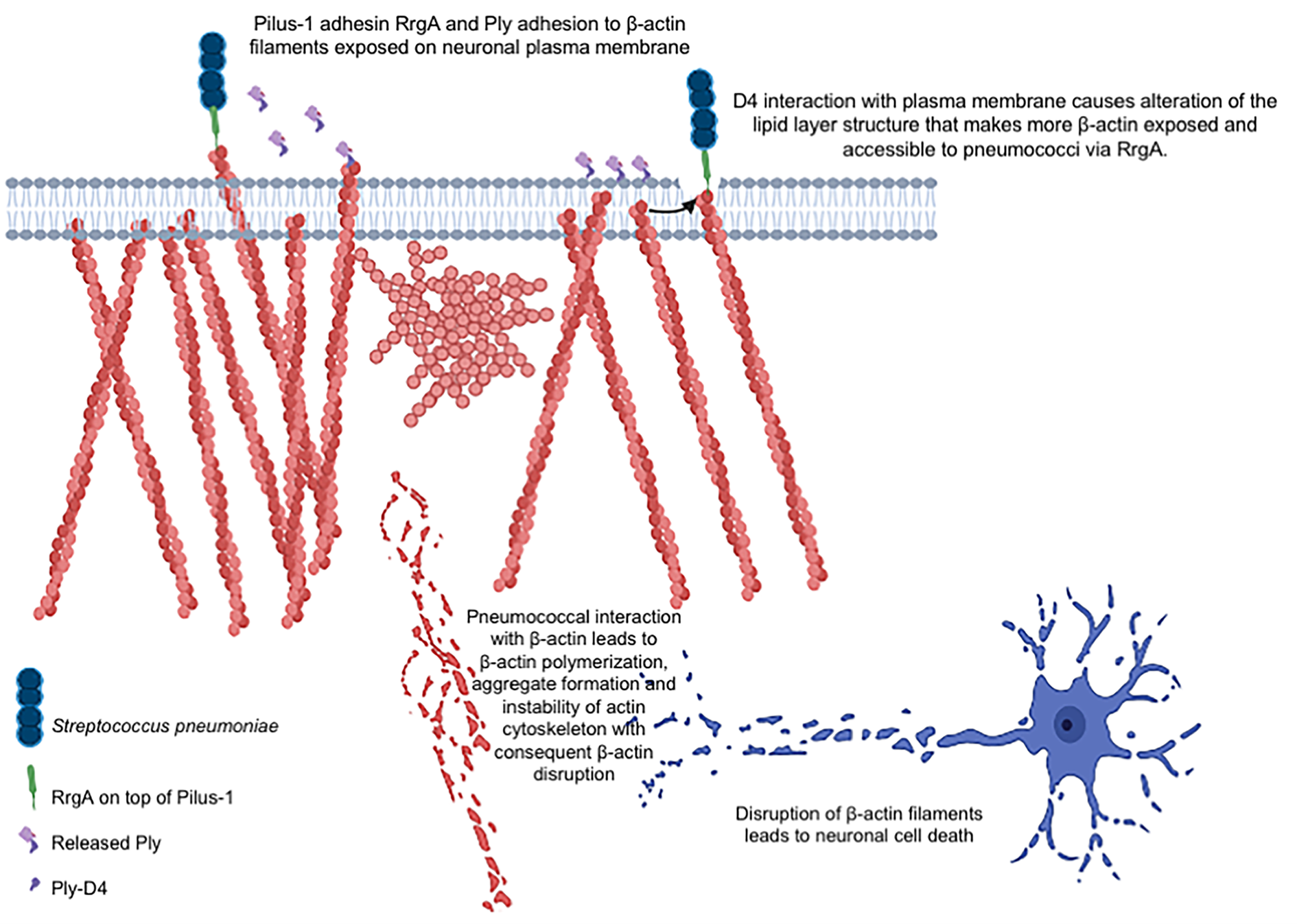

Supplement: S12 Fig — Schematic figure summarizing the molecular mechanism of S. pneumoniae interaction with neurons through released Ply and pilus-1 adhesin RrgA. (TIF) [file ppat.1009432.s012.tif]
