# Supplementary material for: Neuronal death in pneumococcal meningitis is triggered by pneumolysin and RrgA interactions with β-actin
Source: PLoS Pathog. 2021 Mar 24;17(3):e1009432. doi: 10.1371/journal.ppat.1009432 (PMC7990213; doi:10.1371/journal.ppat.1009432)
Supplement: S1 Table — β-actin is shown in bold. (DOCX) [file ppat.1009432.s013.docx]

| **Neuronal proteins bound to RrgA** | **Score** | **Cellular localization** |
| --- | --- | --- |
| Elongation factor Tu | 331.48 | Intracellular (mitochondria) |
| Non-POU domain-containing octamer-binding protein | 620.56 | Intracellular (nucleoplasm) |
| Splicing factor, proline- and glutamine-rich | 511.18 | Intracellular (DNA/RNA-binding) |
| Pre-mRNA-splicing factor ATP-dependent RNA helicase DHX15 | 207.21 | Intracellular (nuclear speckles) |
| Endoplasmic reticulum resident protein 44 | 118.42 | Intracellular (endoplasmatic reticulum) |
| Phospholipase D3 | 77.08 | Intracellular (lysosomes, endosomes, Golgi) |
| Cleavage and polyadenylation specificity factor subunit 7 | 86.06 | Intracellular (nucleoplasm) |
| 5'-nucleotidase domain-containing protein 2 | 111.90 | Intracellular (DNA binding) |
| Heterogeneous nuclear ribonucleoprotein H | 71.52 | Intracellular (nucleoplasm) |
| L-lactate dehydrogenase B chain | 98.82 | Intracellular (cytosol) |
| C-terminal-binding protein 1 | 54.12 | Intracellular (nucleoplasm) |
| DnaJ homolog subfamily C member 10 | 85.61 | Intracellular (chaperone, mitochondria) |
| Heterogeneous nuclear ribonucleoprotein L-like | 72.87 | Intracellular (nucleus) |
| Cleavage and polyadenylation specificity factor subunit 5 | 59.16 | Intracellular (RNA binding) |
| Paraspeckle component 1 | 72.24 | Intracellular (RNA binding) |
| Heterogeneous nuclear ribonucleoprotein H2 | 52.18 | Intracellular (RNA binding) |
| Aflatoxin B1 aldehyde reductase member 2 | 64.57 | Intracellular (Golgi) |
| Thioredoxin-dependent peroxide reductase, mitochondrial | 51.53 | Intracellular (mitochondria) |
| Cleavage and polyadenylation specificity factor subunit 6 | 52.68 | Intracellular (RNA binding) |
| Neurosecretory protein VGF | 129.68 | Secreted |
| Cytosolic purine 5'-nucleotidase | 50.04 | Intracellular (cytosol) |
| Putative RNA-binding protein Luc7-like 2 | 58.74 | Intracellular (RNA binding) |
| Tubulin beta-4B chain | 70.96 | Cytoskeleton |
| Prelamin-A/C | 51.3 | Intracellular (nucleus) |
| Tubulin beta chain | 83.9 | Cytoskeleton |
| Glutamate dehydrogenase 1, mitochondrial | 62.06 | Intracellular (mitochondria) |
| Tubulin alpha-1A chain | 65.09 | Cytoskeleton |
| Dihydropyrimidinase-related protein 4 | 60.43 | Intracellular (cytoplasm) |
| Heat shock cognate 71 kDa protein | 60.08 | Intracellular (nucleus) |
| Secretogranin-1 | 71.2 | Secreted |
| Dihydropyrimidinase-related protein 5 | 80.03 | Intracellular (cytoplasm) |
| Tubulin alpha-1B chain | 64.82 | Cytoskeleton |
| Splicing factor 1 | 51.24 | Intracellular (nucleus) |
| L-lactate dehydrogenase A chain | 62.3 | Intracellular (cytoplasm) |
| ATP-dependent RNA helicase DDX42 | 65.93 | Intracellular (RNA binding) |
| Heterogeneous nuclear ribonucleoprotein L | 125.64 | Intracellular (nucleus) |
| **Beta-actin** | **86.13** | **Cytoskeleton** |
