# Supplementary material for: Neuronal death in pneumococcal meningitis is triggered by pneumolysin and RrgA interactions with β-actin
Source: PLoS Pathog. 2021 Mar 24;17(3):e1009432. doi: 10.1371/journal.ppat.1009432 (PMC7990213; doi:10.1371/journal.ppat.1009432)
Supplement: S2 Table — β-actin is shown in bold. (DOCX) [file ppat.1009432.s014.docx]

| **Neuronal proteins bound to Ply** | **Score** | **Cellular localization** |
| --- | --- | --- |
| Elongation factor Tu | 355.91 | Intracellular (mitochondria) |
| Non-POU domain-containing octamer-binding protein | 778.22 | Intracellular (nucleoplasm) |
| Splicing factor, proline- and glutamine-rich | 650.26 | Intracellular (DNA/RNA-binding) |
| Pre-mRNA-splicing factor ATP-dependent RNA helicase DHX15 | 201.65 | Intracellular (nuclear speckles) |
| Endoplasmic reticulum resident protein 44 | 125.30 | Intracellular (endoplasmatic reticulum) |
| Cleavage and polyadenylation specificity factor subunit 7 | 90.01 | Intracellular (nucleoplasm) |
| Heterogeneous nuclear ribonucleoprotein H | 79.37 | Intracellular (nucleoplasm) |
| Neurosecretory protein VGF | 164.31 | Secreted |
| ATP-dependent 6-phosphofructokinase, muscle type | 84.26 | Intracellular (cytosol, nucleus) |
| Heterogeneous nuclear ribonucleoprotein L-like | 86.22 | Intracellular (nucleoplasm) |
| Creatine kinase U-type, mitochondrial | 55.82 | Intracellular (mitochondria) |
| Glutamate dehydrogenase 1, mitochondrial | 91.65 | Intracellular (mitochondria) |
| Secretogranin-1 | 101.75 | Intracellular (endoplasmatic reticulum) |
| Cleavage and polyadenylation specificity factor subunit 5 | 65.97 | Intracellular (RNA binding) |
| Paraspeckle component 1 | 65.22 | Intracellular (RNA binding) |
| Aflatoxin B1 aldehyde reductase member 2 | 69.72 | Intracellular (Golgi) |
| Cleavage and polyadenylation specificity factor subunit 6 | 59.87 | Intracellular (RNA binding) |
| Cytosolic purine 5'-nucleotidase | 66.97 | Intracellular (cytosol) |
| Putative RNA-binding protein Luc7-like 2 | 61.14 | Intracellular (RNA binding) |
| Tubulin beta-4B chain | 103.96 | Cytoskeleton |
| Prelamin-A/C | 58.51 | Intracellular (nucleus) |
| Tubulin beta chain | 124.54 | Cytoskeleton |
| Tubulin alpha-1A chain | 90.04 | Cytoskeleton |
| Dihydropyrimidinase-related protein 4 | 76.7 | Intracellular (cytoplasm) |
| Dihydropyrimidinase-related protein 5 | 100.75 | Intracellular (cytoplasm) |
| Tubulin alpha-1B chain | 85.79 | Cytoskeleton |
| Splicing factor 1 | 62.5 | Intracellular (nucleus) |
| L-lactate dehydrogenase A chain | 69.29 | Intracellular (cytoplasm) |
| ATP-dependent RNA helicase DDX42 | 79.04 | Intracellular (RNA binding) |
| Heterogeneous nuclear ribonucleoprotein L | 86.22 | Intracellular (nucleus) |
| Zinc finger CCCH-type antiviral protein 1-like | 60.22 | Intracellular (cytoplasm) |
| Cystathionine beta-synthase | 63.36 | Intracellular (nucleus, cytoplasm) |
| Centrosomal protein of 170 kDa | 58.51 | Cytoskeleton |
| 5'-nucleotidase domain-containing protein 2 | 68.43 | Intracellular (cytplasm, nucleus, endoplasmatic reticulum) |
| Heat shock cognate 71 kDa protein | 53.9 | Intracellular (nucleus) |
| L-lactate dehydrogenase B chain | 56.22 | Intracellular (cytoplasm) |
| **Beta-actin** | **106.26** | **Cytoskeleton** |
