# Supplementary material for: Neuronal death in pneumococcal meningitis is triggered by pneumolysin and RrgA interactions with β-actin
Source: PLoS Pathog. 2021 Mar 24;17(3):e1009432. doi: 10.1371/journal.ppat.1009432 (PMC7990213; doi:10.1371/journal.ppat.1009432)
Supplement: S3 Table — β-actin is shown in bold. (DOCX) [file ppat.1009432.s015.docx]

| **Negative control** | **Score** | **Cellular localization** |
| --- | --- | --- |
| Elongation factor Tu | 355.91 | Intracellular (mitochondria) |
| Non-POU domain-containing octamer-binding protein | 778.22 | Intracellular (nucleoplasm) |
| Splicing factor, proline- and glutamine-rich | 439.83 | Intracellular (DNA/RNA-binding) |
| Pre-mRNA-splicing factor ATP-dependent RNA helicase DHX15 | 152.14 | Intracellular (nuclear speckles) |
| Endoplasmic reticulum resident protein 44 | 78.22 | Intracellular (endoplasmatic reticulum) |
| Phospholipase D3 | 37.31 | Intracellular (lysosomes, endosomes, Golgi) |
| Cleavage and polyadenylation specificity factor subunit 7 | 47.70 | Intracellular (nucleoplasm) |
| 5'-nucleotidase domain-containing protein 2 | 74.24 | Intracellular (DNA binding) |
| Heterogeneous nuclear ribonucleoprotein H | 53.09 | Intracellular (nucleoplasm) |
| L-lactate dehydrogenase B chain | 71.13 | Intracellular (cytosol) |
| Neurosecretory protein VGF | 121.05 | Secreted |
| ATP-dependent 6-phosphofructokinase, muscle type | 50.82 | Intracellular (cytosol, nucleus) |
| Heterogeneous nuclear ribonucleoprotein L-like | 53.09 | Intracellular (nucleoplasm) |
| Creatine kinase U-type, mitochondrial | 25.40 | Intracellular (mitochondria) |
| Glutamate dehydrogenase 1, mitochondrial | 62.22 | Intracellular (mitochondria) |
| Secretogranin-1 | 74.12 | Intracellular (endoplasmatic reticulum) |
| Cleavage and polyadenylation specificity factor subunit 5 | 40.59 | Intracellular (RNA binding) |
| Paraspeckle component 1 | 56.10 | Intracellular (RNA binding) |
| Heterogeneous nuclear ribonucleoprotein H2 | 37.30 | Intracellular (RNA binding) |
| Aflatoxin B1 aldehyde reductase member 2 | 52.99 | Intracellular (Golgi) |
| Thioredoxin-dependent peroxide reductase, mitochondrial | 41.85 | Intracellular (mitochondria) |
| Cleavage and polyadenylation specificity factor subunit 6 | 43.58 | Intracellular (RNA binding) |
| Cytosolic purine 5'-nucleotidase | 43.37 | Intracellular (cytosol) |
| Putative RNA-binding protein Luc7-like 2 | 52.84 | Intracellular (RNA binding) |
| Tubulin beta-4B chain | 66.38 | Cytoskeleton |
| Prelamin-A/C | 49.16 | Intracellular (nucleus) |
| Tubulin beta chain | 82.12 | Cytoskeleton |
| Glutamate dehydrogenase 1, mitochondrial | 62.22 | Intracellular (mitochondria) |
| Tubulin alpha-1A chain | 66.82 | Cytoskeleton |
| Dihydropyrimidinase-related protein 4 | 84.8 | Intracellular (cytoplasm) |
| Heat shock cognate 71 kDa protein | 62.49 | Intracellular (nucleus) |
| Dihydropyrimidinase-related protein 5 | 84.8 | Intracellular (cytoplasm) |
| Tubulin alpha-1B chain | 69.88 | Cytoskeleton |
| Splicing factor 1 | 57.59 | Intracellular (nucleus) |
| L-lactate dehydrogenase A chain | 72.1 | Intracellular (cytoplasm) |
| ATP-dependent RNA helicase DDX42 | 79.82 | Intracellular (RNA binding) |
| Heterogeneous nuclear ribonucleoprotein L | 147.03 | Intracellular (nucleus) |
| Zinc finger CCCH-type antiviral protein 1-like | 35.79 | Intracellular (cytoplasm) |
| Cystathionine beta-synthase | 39.93 | Intracellular (nucleus, cytoplasm) |
| Centrosomal protein of 170 kDa | 40.14 | Cytoskeleton |
| **Beta-actin** | **20.41** | **Cytoskeleton** |
