# Supplementary material for: Neuronal death in pneumococcal meningitis is triggered by pneumolysin and RrgA interactions with β-actin
Source: PLoS Pathog. 2021 Mar 24;17(3):e1009432. doi: 10.1371/journal.ppat.1009432 (PMC7990213; doi:10.1371/journal.ppat.1009432)
Supplement: S4 Table — (DOCX) [file ppat.1009432.s016.docx]

| Primer Name | Sequence (5’ – 3’) |
| --- | --- |
| *ply-1* | GCTACCTGTCGCCCTTGCTC |
| *ply-2* | GATATTCTCATTTTAGCCATCTTCTACCTCCTAATAAGTTC |
| *ply-3* | ACTGGATGAATTGTTTTAGGAGAGGAGAATGCTTGCGAC |
| *ply-4* | GCTTGTTTAGCACGGTCGATAAC |
| *kanRfwd* | ATGGCTAAAATGAGAATATC |
| *kanRrev* | CTAAAACAATTCATCCAGT |
