# Supplementary material for: Neuronal death in pneumococcal meningitis is triggered by pneumolysin and RrgA interactions with β-actin
Source: PLoS Pathog. 2021 Mar 24;17(3):e1009432. doi: 10.1371/journal.ppat.1009432 (PMC7990213; doi:10.1371/journal.ppat.1009432)
Supplement: Attachment — Submitted filename: Tabusi et al 2021_Rebuttal letter_Minor Revision.docx [file ppat.1009432.s017.docx]

Federico Iovino, PhD, Asst. Professor

Dept. Neuroscience

Karoliska Insititutet

Biomedicum D07, Solnavägen 9,

SE-17165 Stockholm, SWEDEN

Email: federico.iovino@ki.se

Stockholm, February 12^th^ 2021

To the Editors of *PLOS Pathogens* Carlos Javier Orihuela (Associate Editor), Michael Wessels (Section Editor), Kasturi Haldar (Editor-in-Chief) and Michael Malim (Editor-in-Chief),

Will you please consider our revised version of the manuscript PPATHOGENS-D-20-02733 “Neuronal death in pneumococcal meningitis is triggered by pneumolysin and RrgA interactions with β-actin” by Tabusi M. et al. for publication in *PLOS Pathogens*.

We would like to thank the Editors and the Reviewers for stating that the manuscript is considerably improved. As requested, we have provided additional data to address the experimental concerns raised by reviewer #2. To assist the Editors and reviewers in the assessment of the revised version, all figures containing new data are shown in this point-by-point response to the reviewers below. We have carefully read all comments and have responded, as you will find below.

We now hope that you find our manuscript suitable for publication in *PLOS Pathogens*.

These data were published as a preprint in the bioRxiv (doi: <https://doi.org/10.1101/2020.08.20.258681>), and have not been peer-reviewed elsewhere or are currently under consideration elsewhere. The authors declare no conflict of interest.

With kind regards,

Federico Iovino, PhD, Assistant Professor

**Part I - Summary**

***Reviewer #1***

**This manuscript investigates the role of pneumococcal interactions with neuronal ß actin in the course of brain damage in meningitis. It is well known that there are many mechanisms by which pneumococci can kill cells, including neurons. This manuscript describes a new one. The authors present a very plausible scenario that a pneumococcal pilus brings the bacteria close to the neuronal membrane and this increases the toxicity of the cytotoxin Pln; these steps involve interactions with cell surface ß actin. The experiments follow a logical sequence and the data is solid, well controlled and strongly support the conclusions.**

We thank the reviewer for the positive feedback.
Minor Comments:

**1) Abstract: Mechanisms of neuronal damage in meningitis have been extensively studied in many labs for decades. While this paper presents a new one, it is perhaps inaccurate to begin the abstract with “little is known about mechanisms that lead to neuronal death”.**
The opening sentence of the abstract has been modified with “…little is known about mechanisms of bacterial interaction with neurons leading to neuronal cell death”.
**2) Introduction: Studies from this laboratory group have suggested that pilus is present on only ~20% of clinical pneumococcal strains. This should be made clear and any data indicating if pili are more abundant in meningeal strains should be cited. This would place the new mechanism in the context of the likelihood that in a given patient, the deployment of strategies against the mechanisms of damage shown in this study may not be commonly operative.**
In the Introduction, we have specified (line 50) that the pilus-1 is present in ∼30% of pneumococcal isolates (references 9 and 11 were cited). In addition, we also inserted a sentence in the Discussion stating “Our study therefore provides evidence that all pneumococci, also the strains that do not express pili, can cause neuronal cell damage through the action of Ply” (lines 469-471).
**3) The studies with ß actin are particularly strong and innovative. The careful and detailed microscopy is highly informative and supports the conclusions.**

We thank the reviewer for the positive feedback.

**4) Most of the studies shown are with a neuronal cell line. There are many different neuronal types; which one does this cell represent? In clinical meningitis, it is known that hippocampal neurons are found to be damaged more often than cortical neurons. Does the mechanism described in this paper suggest a reason for this variable susceptibility?**

We thank the reviewer for the comment. For this study we wanted to have an *in vitro* model of neurons (human origin) to setup all infection experiments to study the molecular mechanism of pneumococcal-neuron interaction. For this reason, we used the immortalized SH-SY5Y cell line differentiated into mature neurons. To further confirm the results obtained with these cells, we also used primary neurons isolated from NES (neuro epithelial stem cells). As a follow-up study, we are now investigating pneumococcal interaction with β-actin expressed in the neurons in different brain regions/lobes.

***Reviewer #2***

**The authors showed that neuronal cell death in pneumococcal meningitis is caused by the interaction of the pneumococcal virulence factor Ply and the pilli factor RrgA with beta-actin. Furthermore, they also showed that pneumococci promoted RrgA- and Ply-dependent actin depolymerization, leading to Ca2+ influx and cell death. The results in this paper are too indirect to support their hypothesis, and mechanistic approach to elucidate the significance of Ply- and RrgA- interaction with actin should be done.**

We thank the reviewer for the critical feedback. In the revised manuscript we provide the following additional data:

- Pull-down experiments showing the direct interaction between D4-Ply and β-actin (Figure 3D).
- Neuronal cell death upon treatment with the mutant toxoid PdB (Figure 8H).
- Adhesion assay using neurons infected with pneumococci lacking pilus-1 and Ply (TIGR4∆*rrgA*-*srtD*∆*ply*) in combination with D4 of Ply, showing that in absence of pilus-1 (and therefore RrgA) pneumococci cannot adhere more to neurons even if D4 treatment makes more β-actin exposed on the neuronal plasma membrane (Figure 8F). This finding further supports the conclusion that only in presence of pilus-1 (and RrgA) pneumococci can exploit the increased exposure of β-actin to adhere more to neurons.
- Adhesion assay using neurons infected with pneumococci lacking Ply (TIGR4∆*ply*) in combination with PdB, and TIGR4∆*ply* alone, which shows that the adhesion capacity to neurons is similar using these mutants. This finding further supports the conclusion that PdB alone does not increase the expression of β-actin exposed on the neuronal plasma membrane, therefore no enhanced pneumococcal adhesion is observed (Figure 8G).

***Reviewer #3***

**The authors have taken great care in addressing the concerns of the reviewers, including providing additional data.**

We thank the reviewer for the positive feedback.

**Part II – Major Issues: Key Experiments Required for Acceptance**

**Reviewer #1**

**None.**

**Reviewer #2**

**1. The direct interaction of Ply D4 and actin should be required, and the author should demonstrate whether single amino acid substituted actin-binding deficient Ply-complemented pneumococci fails to increase the amount of β-actin exposed to the cell surface and dampens the RrgA adherence to neuron, Ca2+ influx and cell death without pore-forming activity. It is critical issue to demonstrate their hypothesis directly.**

**Figure 3D.** Detection of D4-Ply and Ni-NTA beads coupled with D4-Ply by Coomassie staining, and western blot analysis performed after co-immunoprecipitation experiment using purified D4-Ply, coupled with Ni-NTA beads, incubated with the purified recombinant proteins β-actin.

We have shown the interaction of D4-Ply with β-actin using pull-down experiment (Figure 3D). Since we do not have antibodies targeting only domain 4 of Ply, and only an antibody targeting the full-length Ply protein, we assessed the proper coupling of Ni-NTA beads with purified D4 though Coomassie staining, using purified D4 (without Ni-NTA beads) as positive control, and to show the correct molecular weight of D4 we showed the ladder as well in the first lane of the gel. We then used Ni-NTA beads coupled with purified D4 to perform pull-down experiment using purified β-actin protein. We show binding of Ni-NTA beads coupled with purified D4 to β-actin (western blot for β-actin, see above the new Figure 3D). As control, we used Ni-NTA beads alone incubated with β-actin, and no β-actin was detected. These results are described in lines 287-288, 289-290, and 429 of the revised manuscript. The use of purified D4 in a pull-down experiment has also been added to the Material and Methods (line 745 of the revised manuscript). Also, the description of the Coomassie staining procedure has been added to the Material and Methods section “Western blot analysis and Coomassie staining” (lines 791 and 809-812 of the revised manuscript).

**Figure 8F.** Adhesion of TIGR4Δ*ply* to untreated and D4-treated neurons and of TIGR4Δ*rrgA*-*srtD*Δ*ply* to D4-treated neurons was calculated by dividing the total number of bacteria in each well for each pneumococcal strain after pneumococcal infection by the total number of adhered bacteria in each well for each pneumococcal strain. Columns represent average values, and error bars represent standard deviations; the graph shows data from n=2 biological replicates (with n=7 and n=5 technical replicates per each biological replicate), * = p<0.05.

In Figure 8F we had already shown that when pneumococci do not have Ply and only have pilus-1 to interact with neuronal β-actin (the case for TIGR∆*ply*), D4 treatment leads to increased β-actin exposed on the plasma, allowing more TIGR∆*ply* pneumococci to adhere to neurons. In addition, we have now included data on adhesion of the double mutant TIGR4∆*rrgA*-*srtD*∆*ply* to neurons pre-treated with D4. We show that in absence of pilus-1 and Ply, even if there is more β-actin exposed on the plasma membrane upon D4 treatment, the capacity of pneumococci to bind to neurons is severely impaired (see above the new Figure 8F). This latest result further highlights the importance of RrgA for pneumococcal adhesion to neuronal β-actin. These results are described in lines 275-279 of the revised manuscript.

**2. Similarly, the author should demonstrate whether single amino acid substituted actin-binding deficient RrgA-complemented pneumococci fails to Ca2+ influx and cell death. It is critical issue to support their hypothesis directly.**

In Figure 7 we had already shown that the non-cholesterol binding and non-pore-forming mutant toxoid PdB, unlike full Ply and D4-Ply, does not increase the exposure of β-actin on the neuronal plasma membrane.

**Figure 8G.** Adhesion of TIGR4Δ*ply* to untreated and PdB-treated neurons was calculated by dividing the total number of bacteria in each well for each pneumococcal strain after pneumococcal infection by the total number of adhered bacteria in each well for each pneumococcal strain. Columns represent average values, and error bars represent standard deviations; the graph shows data from n=3 biological replicates (with n=3 and n=3 technical replicates per each biological replicate), * = p<0.05.

To further strengthen this finding, we performed adhesion assays using untreated and PdB-treated neurons infected with pneumococci lacking Ply (TIGR∆*ply*), so in this case bacteria can only use RrgA on pilus-1 to adhere to neurons. We find that upon PdB treatment there is no increase of β-actin accessible on the plasma membrane, and no difference in pneumococcal adhesion was observed in untreated and PdB-treated neurons (see above new Figure 8G). These results are described in lines 279-282 of the revised manuscript.

**Figure 8H**. Neuronal cell death measured by LDH release in neurons treated with D4, or RrgA, or mutant toxoid PdB, or full Ply, non-treated neurons were used as control; columns represent average values, and error bars represent standard deviations; the graph shows data from n=2 biological replicates (with n=3 technical replicate per each biological replicate), ** = p<0.01, * = p<0.05.

Finally, in Figure 8H we had already shown that D4 alone is enough to induce a significant neuronal cell death. Now, through LDH viability assay we also show that treatment with the mutant toxoid PdB does not induce any significant neuronal cell death (see above the new Figure 8H). These results are described in lines 286-287 of the revised manuscript.

The adhesion assay using pre-treated neurons is described in the Material and Methods (section “Infection of neurons”, lines 540-542 of the revised manuscript).

As a follow-up study (and follow-up manuscript), we are now investigating more in depth the molecular mechanism on a protein and, specifically on a single-amino acid level, which parts of the Ply and RrgA domains are involved in the interaction with β-actin. Our ultimate goal for the next study is to precisely characterise this interaction, in order to develop an approach to block pneumococcal-neuron interaction *in vivo*.

**Reviewer #3**

**None.**

**Part III – Minor Issues: Editorial and Data Presentation Modifications**

**Reviewer #1**

**Listed in Part 1**

We have addressed the comments of reviewer #1 in Part I (see above pages 1 and 2).

**Reviewer #2**

**1. Quality of images are too poor.**

All immunofluorescence microscopy images taken using the high-resolution Delta Vision Elite Imaging System were obtained applying deconvolution with Softworx imaging program (Applied Precision) which further improves the contrast and resolution and reduces the background noise of images captured by the sCMOS camera. The same microscope setup was also used in other publications from our group cited in the manuscript (references 9 and 16). Furthermore, in Figure 5 we also applied a 3D reconstruction model to visualize pneumococcal interaction with neuronal β-actin *ex vivo*.

Super-resolution STED microscopy was used to have a direct evidence of disruption of neuronal β-actin filaments upon pneumococcal infection. To avoid signal overlap between different fluorophores, we decided to exclusively focus on β-actin using only one primary antibody and one secondary antibody. This microscopy technique provides a much higher resolution (super resolution) than confocal systems since it overcomes the diffraction limited resolution of confocal microscopes. The same STED microscopy setup was also used in another publication from our group cited in the manuscript (reference 16).

The resolution in all figures respect the *PLOS Pathogens* figure guidelines.

**2. Fig. S12 should be included in main text**.

Supplementary Figure S12, the cartoon figure that provides an overall overview of the molecular mechanism of pneumococcal-neuron interaction, is cited in the Discussion.

**Reviewer #3**

**None.**
